# Refractory Thrombotic Thrombocytopenic Purpura to Therapeutic Plasma Exchange

**DOI:** 10.7759/cureus.29562

**Published:** 2022-09-25

**Authors:** Amy Kiamos, Kimberly Boldig, Pramod Reddy

**Affiliations:** 1 Internal Medicine, University of Florida College of Medicine – Jacksonville, Jacksonville, USA

**Keywords:** thrombocytopenia, therapeutic plasma exchange (tpe), plasmapheresis, microangiopathic hemolytic anemia, caplacizumab, ttp, thrombotic thrombocytopenic purpura

## Abstract

Thrombotic thrombocytopenic purpura (TTP) is a rare, potentially fatal hematologic disorder characterized by microangiopathic hemolytic anemia, thrombocytopenia, and varying signs of visceral ischemia secondary to microvascular thrombosis. TTP is caused by a severe deficiency of ADAMTS13, a protease enzyme responsible for cleaving von Willebrand-factor (vWF) multimers. First-line therapy with plasmapheresis has increased survival rates immensely; however, there are few reported cases that are refractory to standardized treatment. We describe two cases of refractory TTP successfully managed with the addition of caplacizumab, an anti-von Willebrand factor immunoglobulin fragment that inhibits the interaction of vWF multimers with platelets.

## Introduction

Thrombotic thrombocytopenic purpura (TTP) is a life-threatening, hematologic emergency that is easily missed and requires a high index of suspicion for diagnosis. TTP is characterized by microangiopathic hemolytic anemia (MAHA), thrombocytopenia, and varying signs of visceral ischemia caused by microvascular thrombosis [1]. TTP is caused by a severe deficiency of ADAMTS13 (a disintegrin and metalloproteinase with a thrombospondin type 1 motif, member 13), a von Willebrand-factor (vWF) cleaving metalloprotease responsible for reducing the size of vWF multimers [2]. In TTP, a severe ADAMTS13 deficiency (acquired or inherited) leads to the loss of vWF size regulation, subsequently resulting in abnormally large vWF multimers that are unable to be broken down. These large vWF multimers aggregate with platelets leading to thrombocytopenia and microvascular thrombosis causing varying symptoms of end-organ ischemia [2]. Red blood cells are mechanically sheared due to obstructing thrombi in the lumen, which ultimately leads to hemolytic anemia with schistocytes visible on peripheral smears [3].

TTP has an incidence of one case per million per year and a mortality rate of up to 20% despite treatment [1]. Hereditary TTP is caused by ADAMTS13 genetic mutations leading to severe ADAMTS13 deficiency, whereas acquired TTP is caused by autoantibodies against ADAMTS13 [4]. TTP is traditionally known for the clinical pentad of fever, MAHA, thrombocytopenia, neurological symptoms, and renal dysfunction; however, the full pentad is seen in less than 10% of cases [1,5]. Symptoms driven by end-organ ischemia in TTP are extremely variable in presentation and severity [1,5-6]. The diagnosis of TTP requires a high clinical index of suspicion with prompt initiation of therapeutic management in order to reduce morbidity and mortality. Scoring systems, such as Coppo’s scoring system and the PLASMIC score, can help determine the pretest probability of TTP in order to guide management. It is critical to quickly recognize patients with suspected TTP to begin life-saving treatment; survival is less than 10% without prompt initiation of plasmapheresis [5]. Here we present two cases of refractory TTP to standard treatment of plasmapheresis.

## Case presentation

Case 1

A 23-year-old female with no significant past medical history presented to the emergency department (ED) with a six-day history of nausea and vomiting. She reported multiple episodes of non-bloody, non-bilious emesis associated with abdominal pain, new-onset headaches, and intermittent “spots” in her visual fields. On admission, the patient was afebrile and hemodynamically stable. Her physical exam was unrevealing.

Initial lab work was significant for marked thrombocytopenia of 22 thou/mm^3^, normocytic anemia, and normal renal function (Table 1). Additional labs were suspicious for hemolysis with lactate dehydrogenase (LDH) of 522 IU/L, haptoglobin of <10 mg/dL, and a reticulocyte count of 7.9%. The peripheral blood smear noted a moderate amount of schistocytes. Given the evidence of active hemolysis and significant severe thrombocytopenia, there was increased concern for TTP. The PLASMIC score was calculated at 7 points, indicating a high risk of TTP. Subsequent ADAMTS13 activity testing returned at 16% (normal >60%) and the ADAMTS13 inhibitor at 63% (normal <30%). Other differential diagnoses, including atypical hemolytic uremic syndrome and autoimmune hemolytic anemia, were excluded based on normal renal function, unremarkable urinalysis, negative direct antiglobulin test, and negative direct Coomb’s test for C3B/C3D. She tested negative for human immunodeficiency virus (HIV), hepatitis, and respiratory viruses.

**Table 1:** Laboratory Results for Case 1

Lab test	Result	Reference Range
Platelets (plt)	22 (thou/mm^3^)	150-450 (thou/mm^3^)
White Blood Cell (WBC)	11.55 (thou/mm^3^)	3.4-10.8 (thou/mm^3^)
Hemoglobin (Hgb)	8.6 (g/dL)	11.1-15.9 (g/dL)
Mean Corpuscular Volume (MCV)	86 (fL)	79-97 (fL)
Creatinine (Cr)	0.66 (mg/dL)	0.57-1 (mg/dL)
Lactate Dehydrogenase (LDH)	522 (IU/L)	126-266 (IU/L)
Haptoglobin	<10 (mg/dL)	30-200 (mg/dL)
Reticulocyte Count	7.9%	0.5-1.5%
Immature Platelet Fraction (IPF)	4.7%	1.7-7%
Absolute Reticulocyte Count	0.2571 (10^6^/mm^3^)	0.0225-0.0945 (10^6^/mm^3^)
ADAMTS13 Activity	16%	>60%
ADAMTS13 Inhibitor	63%	<30%
Erythrocyte Sedimentation Rate (ESR)	40 (mm/hr)	0-20 (mm/hr)
C-reactive Protein (CRP)	7.3 (mg/L)	0-5 (mg/L)
Prothrombin Time (PT)	14.9 (sec)	9.4-12.5 (sec)
International Normalized Ratio (INR)	1.3	0.8-1.1
Partial Thromboplastin Time (PTT)	33.6 (sec)	25-37 (sec)
Ferritin	508 (ng/mL)	15-150 (ng/mL)
Iron, Serum	85 (ug/dL)	32-159 (ug/dL)
Iron Saturation	26%	20-55%
Total Iron-Binding Capacity (TIBC)	328 (mcg/dL)	261-390 (mcg/dL)
Transferrin	258 (mg/dL)	200-360 (mg/dL)
Folate	8.4 (ng/mL)	4.2-19.9 (ng/mL)
Vitamin B12	434 (pg/mL)	232-1,245 (pg/mL)

The patient was started on therapeutic plasma exchange (also known as plasmapheresis) in addition to high-dose steroids with prednisone 100 mg daily (1 mg/kg). She underwent four sessions of plasmapheresis with subsequent recovery of the platelet count from 20 to 220 thou/mm^3^ (Table 2). Plasmapheresis was discontinued at this time; however, she began to have an acute drop in platelet count to 34 thou/mm^3^ and was thus started on her fifth round of plasmapheresis. She did not receive rituximab at this time given that her ADAMTS13 activity was greater than 10%. She received three more rounds of plasmapheresis (a total of eight sessions) with an improvement of her platelet count to 266 thou/mm^3^. However, she had another decrease in platelet count two days later to 53 thou/mm^3^. Caplacizumab was then initiated with 10 mg intravenously (IV), followed by 10 mg subcutaneously (SQ) on the first day. She was continued on caplacizumab 10 mg SQ daily and received two more rounds of plasmapheresis (total of 10 sessions) with her platelet count recovering to 255 thou/mm^3^. Plasmapheresis was stopped at this time, and she continued with caplacizumab 10 mg SQ daily. Her platelet count continued to increase and stabilize over the next couple of days, eventually reaching 477 thou/mm^3^.

**Table 2:** Plasmapheresis and Caplacizumab Timeline Case 1

Day	Platelet Count (thou/mm^3^)	Therapeutic Management Timeline	Total Sessions of Plasmapheresis
Day 1	22		
Day 2	27		
Day 3	23	Plasmapheresis started	1
Day 4	72		2
Day 5	125		3
Day 6	169		4
Day 7	220	Plasmapheresis stopped	
Day 8	150		
Day 9	34	Plasmapheresis restarted	5
Day 10	81		6
Day 11	171		7
Day 12	257		8
Day 13	266	Plasmapheresis stopped	
Day 14	165		
Day 15	53	Plasmapheresis restarted and caplacizumab started	9
Day 16	132		10
Day 17	255	Plasmapheresis stopped	
Day 18	294		
Day 19	348		
Day 20	447		

She was discharged with a prolonged prednisone taper and caplacizumab 10 mg SQ daily with a presumed stop date of 30 days after the last session of plasmapheresis. She had a close follow-up with hematology with weekly complete blood cell counts to monitor for thrombocytopenia. At outpatient follow-up, her platelet count remained stable ranging between 390 and 450 thou/mm^3^.

Case 2

A 59-year-old female with a past medical history of refractory TTP and prior cerebrovascular accidents presented to the ED after thrombocytopenia was noted on routine blood work. She admitted to a headache but denied any other complaints. She was first diagnosed with TTP in her early 20s and has had numerous relapses since diagnosis. She was previously managed with plasmapheresis and rituximab due to the recurrent nature of her disease. Prior to her presentation, she received treatment for TTP at an outside hospital, with four sessions of plasmapheresis in addition to rituximab.

Upon presentation, she was afebrile and hemodynamically stable with an unremarkable physical exam. Laboratory workup was significant for thrombocytopenia of 63 thou/mm^3^, normocytic anemia, and schistocytes on the peripheral blood smear (Table 3). Subsequent ADAMTS13 activity testing returned at 23% (normal >60%) and ADAMTS13 inhibitor at 54% (normal <30%). Of note, her ADAMTS13 activity on prior admission was <10%.

**Table 3:** Laboratory Results for Case 2

Lab test	Result	Reference Range
Platelets (plt)	63 (thou/mm^3^)	150-450 (thou/mm^3^)
White Blood Cell (WBC)	9.13 (thou/mm^3^)	3.4-10.8 (thou/mm^3^)
Hemoglobin (Hgb)	12.3 (g/dL)	11.1-15.9 (g/dL)
Mean Corpuscular Volume (MCV)	83.1 (fL)	79-97 (fL)
Creatinine (Cr)	0.94 (mg/dL)	0.57-1 (mg/dL)
Lactate Dehydrogenase (LDH)	467 (IU/L)	126-266 (IU/L)
Haptoglobin	83 (mg/dL)	30-200 (mg/dL)
Reticulocyte Count	1.6%	0.5-1.5%
Immature Platelet Fraction (IPF)	7.3%	1.7-7%
Absolute Reticulocyte Count	0.0741 (10^6^/mm^3^)	0.0225-0.0945 (10^6^/mm^3^)
ADAMTS13 Activity	23%	>60%
ADAMTS13 Inhibitor	54%	<30%
Prothrombin Time (PT)	11.2 (sec)	9.4-12.5 (sec)
International Normalized Ratio (INR)	1.0	0.8-1.1
Partial Thromboplastin Time (PTT)	32 (sec)	25-37 (sec)
Ferritin	186 (ng/mL)	15-150 (ng/mL)
Iron, Serum	61 (ug/dL)	32-159 (ug/dL)
Iron Saturation	20%	20-55%
Total Iron-Binding Capacity (TIBC)	307 (mcg/dL)	261-390 (mcg/dL)
Transferrin	242 (mg/dL)	200-360 (mg/dL)
Folate	16.4 (ng/mL)	4.2-19.9 (ng/mL)
Vitamin B12	531 (pg/mL)	232-1,245 (pg/mL)

The patient was treated at our institution with five sessions of plasmapheresis and high-dose steroids with methylprednisolone 100 mg daily. After five courses of plasmapheresis, her platelets improved to 170 thou/mm^3^ (Table 4). Due to her previous TTP history and high relapse rate, caplacizumab was started with 10 mg IV, followed by 10 mg SQ daily for 30 days after the last session of plasmapheresis. She was discharged the next day with a platelet count of 195 thou/mm^3^. At outpatient follow-up with hematology, her platelet counts remained stable.

**Table 4:** Plasmapheresis and Caplacizumab Timeline Case 2

Day	Platelet Count (thou/mm^3^)	Therapeutic Management Timeline	Total Sessions of Plasmapheresis
Day 1	63	Plasmapheresis started	1
Day 2	92		2
Day 3	138		3
Day 4	170	Caplacizumab started	4
Day 5	195	Plasmapheresis stopped	5
Day 6	362		

## Discussion

TTP is a challenging diagnosis to confirm due to varying clinical presentations and overlap with other microangiopathic hemolytic anemias such as hemolytic uremic syndrome. A severe deficiency of ADAMTS13 (activity level of less than 10%) is characteristic of the diagnosis of TTP. Obtaining an ADAMTS13 level is often untimely; therefore, it should not be used to decide whether to initiate or withhold treatment [5]. In addition, a study from the Harvard TMA Research Collaborative registry found that 73% of TTP patients had ADAMTS13 activity of more than 10% [7]. The deficiency of ADAMTS13 activity is the cornerstone of the pathogenesis of TTP; however, the decision to initiate life-saving treatment for TTP is based on clinical judgment.

Plasma exchange has been the cornerstone of TTP treatment, as it increases survival and is relatively well-tolerated. Plasma exchange is the recommended treatment modality of TTP and should be initiated if there is a high index of suspicion for TTP [8]. Plasma exchange works by separating the plasma component from whole blood through centrifugation or membrane filtration. Centrifugation spins the blood and then separates blood components by gravity while filtration uses a membrane filter to separate plasma components based on molecular weight [8]. In the management of TTP, plasma exchange removes ADAMTS13 antibodies and can help replace normal factors if they are deficient in the patient’s plasma [9]. Plasma exchange is usually performed daily until organ dysfunction has resolved and platelet count stabilizes for at least two days [10]. Corticosteroids have also been part of the foundational treatment in TTP; they are used to target the autoimmune pathogenesis of the disease. Although they are widely accepted for their role in the treatment of TTP, their efficacy remains low [10].

Plasma exchange has made remarkable strides in the treatment of TTP by increasing survival in patients from <10% to 80% [11-12]. Despite the significant improvement in outcomes, refractory or relapsing TTP can occur. Rituximab has been shown to induce remission in >90% of patients [11-13]. Rituximab acts on B-lymphocytes, which produce the antibody to ADAMTS13, subsequently inhibiting antibody production and increasing ADAMTS13 activity [14]. Currently, rituximab has three general recommended indications for use in TTP: the treatment of an acute episode with plasma exchange and corticosteroids, treatment of refractory disease, and prophylaxis in asymptomatic patients with severe ADAMTS13 deficiency [12-15]. More specifically, it has been proposed to use rituximab in cases of ADAMTS13 levels <10% [11,15]. Some believe there is a correlation between relapse rate with lower ADAMTS13 activity level; however, this association is still uncertain [16]. Rituximab use for this indication does not necessarily provide a clear clinical benefit. Additionally, rituximab use does prevent the risk of future relapse [17]. Our first case identified an ADAMTS13 activity level of 16% with continued refractory TTP. This demonstrates the shortcomings of solely utilizing ADAMTS13 levels to guide treatment. Rituximab was also not used at our institution in our case of refractory TTP. This may represent a treatment option with decreased toxicity and cost that may be associated with rituximab use, as our patient was successfully treated with plasma exchange and caplacizumab alone.

Caplacizumab is a newer therapeutic option for the treatment of refractory TTP. Caplacizumab is an anti-vWF immunoglobulin fragment that inhibits vWF multimer interaction with platelets [18]. The initiation of caplacizumab immediately arrests microthrombosis formation and platelet consumption [19]. In two randomized controlled studies, caplacizumab, in addition to standard treatment, resulted in faster time to platelet level response (39% reduction), reduced recurrence of TTP (67% reduction), less plasmapheresis requirement, and shorter hospitalization [18-19]. Caplacizumab has been shown to reduce cases of refractory TTP, death during treatment, and overall mortality in an integrated analysis of 220 patients with TTP [20]. Real-world experience with caplacizumab to treat acute TTP has shown favorable outcomes that are similar to randomized controlled studies [17]. Caplacizumab has a side effect of increased bleeding risk; however, bleeding tends to be minor [17]. With such impressive results, caplacizumab sparks a debate about reconsidering standardized treatment for TTP. Currently, caplacizumab is not considered the first-line treatment for patients with TTP due to its expensive cost. Hopefully, with more clinical experience and more successful patient outcomes, this medication can become more readily accessible and affordable to all patients.

## Conclusions

Here, we presented two cases of refractory TTP that were successfully treated with caplacizumab. Our first case is unique given that the patient was successfully treated with caplacizumab and plasmapheresis alone. Our second case represents a patient with refractory TTP despite prior treatment with plasmapheresis and rituximab who was also successfully treated with caplacizumab and plasmapheresis. Both of these case presentations identify refractory TTP cases despite ADAMTS13 levels >10%. Therapeutic plasma exchange remains the gold-standard treatment for TTP. Refractory cases are typically managed with the addition of rituximab to plasmapheresis. Recent trials have shown promising outcomes of caplacizumab’s role in refractory TTP. With further understanding of caplacizumab’s efficacy and real-world experience of successful outcomes, the above cases add to the growing literature and debate on reconsidering standardized treatment for TTP.
